# From Climate Endgame to Climate Long Game

**DOI:** 10.1073/pnas.2214975119

**Published:** 2022-11-02

**Authors:** Avit Bhowmik, Mark S. McCaffrey, Juliette Rooney Varga

**Affiliations:** ^a^Centre for Research on Sustainable Societal Transformation, Karlstad University, 651 88 Karlstad, Sweden;; ^b^Risk and Environmental Studies, Karlstad University, 651 88 Karlstad, Sweden;; ^c^The Long Game/Independent Scholar, 71110 Marcigny, France;; ^d^Climate Change Initiative, University of Massachusetts Lowell, Lowell, MA 01854;; ^e^Rist Institute for Sustainability and Energy, University of Massachusetts Lowell, Lowell, MA 01854

Kemp et al.’s (1) “Climate Endgame” reaffirms the imperative of taking the potentially existential threat of human-induced climate change seriously rather than simply dismissing it as “gloom and doom.” They put forward a research agenda for integrated climate catastrophe assessment, emphasizing the need for in-depth understanding of extreme climate change dynamics and mass human morbidity, mortality, and social instability. While such a research agenda is indeed needed, it also risks portraying climate change as unsolvable and inevitable, raising the potential for fear and hopelessness, and may even trigger inaction (2–4). It lacks a participatory action research (5) framework that is needed to enable communities to innovate and deploy effective climate solutions customized to their needs and capacities that can be scaled when actively shared with others (6).

We agree with the authors that much of the climate change science literature underestimates the potential for catastrophic impacts and agree that it is important to investigate worst-case scenarios for strong policy measures (1). However, as the authors also stated, it is vital to convey that dangerous climate change is already here, and the action taken today will determine how much worse or better it will be in the future. Hence, a participatory action research agenda emphasizing appropriate individual and collective agency at scale, thereby helping counter fear and hopelessness, should be of the highest priority.

Our ability to survive and potentially thrive in the future will depend on decisions made at every scale of society, but particularly at a “glocal” scale (roughly 10,000 to a million people) where adaptation and mitigation can be effectively deployed and benefits maximized (5). Neither macro nor micro, this meso scale—the size of many local governments (including school districts) and indigenous people bodies—is “just right” for community and capacity building. It's small enough that people can know and trust each other and implement culturally and economically sensible projects, but large enough to trigger meaningful climate action.

To that end, we offer an alternative research agenda, “The Climate Long Game,” that has greater potential to enhance humanity’s resilience than another Intergovernmental Panel on Climate Change report or even a global assessment model on catastrophe—regardless of how close to the ultimate endgame humanity is.

Focusing on the glocal-scale sweet spot, where individuals living their everyday lives intersect with humanity’s planetary scale, our proposed agenda has the following key elements:

• A global database of glocal sweet spots/communities (e.g., ∼100,000 segments of <100,000 people), drawing from open spatial demographic (6) and environmental data to provide public access to relevant, easily understood information, that is, environmental and social strengths, weaknesses, threats and opportunities, administrative boundaries, and key organizations;
• Pedagogical and digital tools including role-playing, simulations, and gamification (7–9) to directly inform and empower those communities, including local governments, schools, and civil society; and
• Direct support and mutual aid at the glocal scale to reduce risks, increase survivability, and facilitate dynamic inclusiveness and deliberate democracy (10).
